# Health-related quality of life and mental health in the medium-term aftermath of the *Prestige *oil spill in Galiza (Spain): a cross-sectional study

**DOI:** 10.1186/1471-2458-7-245

**Published:** 2007-09-17

**Authors:** José Miguel Carrasco, Beatriz Pérez-Gómez, Maria José García-Mendizábal, Virginia Lope, Nuria Aragonés, Maria João Forjaz, Pilar Guallar-Castillón, Gonzalo López-Abente, Fernando Rodríguez-Artalejo, Marina Pollán

**Affiliations:** 1 Environmental and Cancer Epidemiology Unit, National Center for Epidemiology, Carlos III Institute of Health, Madrid, Spain; 2 Research Suppport Unit, La Mancha Centro Hospitalary Complex, Alcazar de San Juan, Ciudad Real, Spain; 3 Consortium for Biomedical Research in Epidemiology & Public Health (CIBER en Epidemiología y Salud Pública – CIBERESP-), Spain; 4 National School of Public Health. Carlos III Institute of Health, Madrid, Spain; 5 Department of Preventive Medicine and Public Health, School of Medicine, Universidad Autónoma de Madrid, Madrid, Spain

## Abstract

**Background:**

In 2002 the oil-tanker *Prestige *sank off the Galician coast. This study analyzes the effect of this accident on health-related quality of life (HRQoL) and mental health in the affected population.

**Methods:**

Using random sampling stratified by age and sex, 2700 residents were selected from 7 coastal and 7 inland Galician towns. Two exposure criteria were considered: a) residential exposure, i.e., coast versus interior; and b) individual exposure-unaffected, slightly affected, or seriously affected-according to degree of personal affectation. SF-36, GHQ-28, HADS and GADS questionnaires were used to assess HRQoL and mental health. Association of exposure with suboptimal scores was summarized using adjusted odds ratios (OR) obtained from logistic regression.

**Results:**

For residential exposure, the SF-36 showed coastal residents as having a lower likelihood of registering suboptimal HRQoL values in physical functioning (OR:0.69; 95%CI:0.54–0.89) and bodily pain (OR:0.74; 95%CI:0.62–0.91), and a higher frequency of suboptimal scores in mental health (OR:1.28; 95%CI:1.02–1.58). None of the dimensions of the other questionnaires displayed statistically significant differences.

For individual exposure, no substantial differences were observed, though the SF-36 physical functioning dimension rose (showed better scores) with level of exposure (91.51 unaffected, 93.86 slightly affected, 95.28 seriously affected, p < 0.001).

**Conclusion:**

Almost one and a half years after the accident, worse HRQoL and mental health levels were not in evidence among subjects exposed to the oil-spill. Nevertheless, some of the scales suggest the possibility of slight impact on the mental health of residents in the affected areas.

## Background

On November 13, 2002, the petrol-tanker, *Prestige*, carrying 77,033 tons of fuel, sank 260 km off the Galician coast. This led to a major spill, with the first oil-laden tide arriving on the Galician coast on November 16 and spreading along the entire Cantabrian shoreline over the following weeks [1].

The fuel spilled was type M100 or No.6 (as per Russian and Anglo-American classifications, respectively) [2], which is mainly used as an industrial fuel. Due to its high density and viscosity and negligible solubility and volatility, it tends to persist in the environment, and manual removal is required to increase the efficacy of clean-up procedures [3]. The International Agency for Cancer Research has classified it as a possible human carcinogen (category 2B) [4].

The oil slick affected most of the Galician seaboard, and led to a ban on fishing and shellfishing. The accident made a great impression on Spanish public opinion and thousands of persons headed for Galicia (Galiza) to work as volunteers in the clean-up operation. The Galician seamen sailed out to sea to mop up the fuel before it arrived onshore and the authorities engaged staff, preferably from among the local ranks of the unemployed, to remove the oil.

This is not the first time that the Galician coast has been affected by oil-spills. Since 1970 it has been the victim of five major disasters of this type [5]. Nevertheless, the sheer scale of the *Prestige *accident, with successive waves of oil coming ashore over a period of weeks, and the serious environmental and economic consequences [3], led to the greatest ecological catastrophe in the region's history [6].

Several studies have reported the prevalence of acute health problems among contract workers and volunteers involved in the *Prestige *oil-spill clean-up, both in Galiza and other areas across the north-west of Spain [7-9]. In the mid and long term, health problems could be different to those encountered in the acute phase and are, in all likelihood, not limited to persons directly implicated in the clean-up [10]. In this respect, the literature has described a rise in social upheavals and mental health disorders among victims of both natural and technological or man-made disasters [11-14].

Proper assessment of the health consequences for persons affected by catastrophes ought to take all health dimensions into account. HRQoL is a multidimensional construct, which is determined, not only by health status, but also by each person's subjective perception of his/her physical, psychological, social, economic and political environment [15-17]. Thus HRQoL might serve to assess the global health impact of catastrophes.

Accordingly, this study examines the association between the *Prestige *oil-spill and the HRQoL and mental health of the general population of Galiza, assessed sixteen months after the accident.

## Methods

### Study participants

The study participants were persons aged 18–60 years residing in 7 Galician coastal towns that received the brunt of the oil (Corcubión, Carnota, Fisterra, Laxe, Camariñas, Cee and Muxía) and in another 7 towns inland (Frades, Masía, Trazo, Tordoia, Cerceda, Oroso and Ordes) that would serve as reference, because they shared sociodemographic and economic characteristics with the coastal towns affected by the *Prestige *spill.

The sample size was calculated to show odds ratios (OR) ≥ 2, with a power of 80%, assuming that the prevalence of subjects with suboptimal values for the dimension of greatest interest (mental health) would be 2%. We decided to re-interview this population in the future and the sample size was therefore increased by 15% to take possible losses. Under these conditions, the size of the sample totaled 1350 subjects in each geographic area (coast and interior). Study subjects were selected from municipal electoral rolls, using random sampling stratified by age, sex and town. Three equivalent randomized samples of 2700 subjects each, were selected. One of the three was considered the main sample, and each subject was assigned two substitutes with similar characteristics, drawn from the other two samples. Hence, 1510 participants (56%) were drawn from the first list, 807 substitutes (30%) from the second list and 383 (14%) from the third. The main reasons for replacing the person of first choice were: flawed census data (17.6%); impossibility of contact (15.1%); and refusal to respond (11.4%) [see Additional file 1].

### Study variables

Data were collected by home-based face-to-face interviews conducted by trained interviewers from March 22 through April 23, 2004. The questionnaire included three modules: a) basic sociodemographic variables (sex, age, educational level, and occupation), job security (work status, and financial coverage), lifestyle (alcohol, tobacco and coffee consumption, and hours of sleep), self-reported morbidity, use of healthcare services, and specific oil-spill exposure questions; b) participation in oil spill clean-up tasks; and c) HRQoL assessed with the 36-item Short Form Health Survey (SF-36) [18], and mental health status assessed with the General Health Questionnaire (GHQ-28) [19], Goldberg Anxiety and Depression Scale (GADS) [20], and Hospital Anxiety Depression Scale (HADS) [21]. These questionnaires were chosen as they had been validated for the Spanish population and been widely used in clinical and population-based studies.

The SF-36 includes information on 8 dimensions of HRQoL: physical functioning; role-physical; bodily pain; general health; vitality; social functioning; role-emotional; and mental health. Each dimension is measured on a continuous scale from 0 (worst value) to 100 (best value) with a difference ≥ 3 being deemed clinically relevant [18,22,23]. A dichotomous variable was defined for each dimension (suboptimal versus optimal score) with the 25^th ^percentile of study subjects' scores taken as the cut-off point.

The GHQ-28 measures the following 4 health dimensions: somatic symptoms; anxiety and insomnia; social dysfunction; and severe depression. We used the bimodal response scale known as the GHQ (0-0-1-1) [24], taking 4/5 as the cut-off to define suboptimal health [19].

Both the HADS and GADS questionnaires consist of an anxiety and a depression subscale. The HADS was designed as an instrument to detect depression and anxiety disorders in a non-psychiatric hospital framework, and defines a probable case as anyone who scores over 7 and less than 11 points, and a confirmed case as anyone who scores 11 or more points on each subscale [21,25,26].

The GADS is formed by two subscales, each structured into 4 screening questions and 5 probe questions. The cut-off points set for analysis were >4 for the anxiety scale and >3 for the depression scale [20,27-29].

Two different criteria of oil-spill exposure were defined: a) residential exposure, with coastal residents defined as exposed, and inland residents as unexposed; and, b) individual exposure, classified in accordance with the scores for the following items: use of coasts affected (0 = no; 1 = no for respondent but yes for cohabitant under same roof; 2 = yes); having worked on clean-up tasks (0 = no; 1 = yes); direct contact with oil through fishing, farming or leisure activities (0 = no; 1 = occasionally through leisure or work; 2 = repeatedly through leisure or work; 3 = repeatedly through leisure and work); oil-spill damage to properties (0 = no; 1 = slightly; 2 = seriously); damage to usual fishing or shellfishing areas (0 = no; 1 = some areas; 2 = practically all areas); respondent's commercial or leisure activities affected (0 = no; 1 = leisure; 2 = commercial, fishing or farming); and, finally, degree to which usual summer holiday beaches were affected (0 = no; 1 = yes, but not enough to make him/her desist from going there; 2 = yes, rendered unfit for swimming). By summing the scores, we obtained a scale with a range of 0 to 12, on the basis of which persons with 0 points were rated as "unaffected", those with 1–5 points as "slightly affected", and those with ≥ 6 points as "seriously affected".

### Statistical analysis

Differences in proportions were tested with the Chi-squared and Fisher's exact tests, and differences in means with the Student's t test, analysis of variance, and test for trend. The association between oil-spill exposure and suboptimal HRQoL and mental health scores was summarized with OR and 95% confidence intervals obtained by logistic regression, adjusted for age, sex, work status, education, smoking, number of hours of sleep daily, number of self-reported chronic diseases, as well as the other type of oil-spill exposure.

Analyses were performed with the Stata 8.2. software package [30].

## Results

Table 1 lists the characteristics of the study participants according to residential and individual exposure. Due to the design, the age- and sex-based distribution of subjects was similar between coastal and inland towns. On the coast, however, there was a higher proportion of persons who: had no formal education; were unemployed or first-time job-seekers; and were smokers. In addition, these subjects reported sleeping fewer hours and had a higher prevalence of diabetes and asthma.

**Table 1:** Characteristics of the study participants according to residential and individual exposure to the *Prestige *oil-spill

	**Residential exposure**					**Individual exposure**						
		
	**Interior (n = 1350)**		**Coast (n = 1350)**			**Unaffected (n = 865)**		**Slightly affected (n = 1408)**		**Seriously affected (n = 427)**		
		
	**N**	**%**	**N**	**%**	**p**	**N**	**%**	**N**	**%**	**N**	**%**	**p**
				
**Sex**					0.878							<0.001
Men	684	50.7	688	51.0		437	50.5	671	47.7	264	61.8	
Women	666	49.3	662	49.0		428	49.5	737	52.3	163	38.2	
												
**Age (years)**					0.948							<0.001
18–29	408	30.2	405	30.0		213	24.6	469	33.3	131	30.7	
30–44	482	35.7	477	35.3		283	32.7	514	36.5	162	37.9	
45–60	460	34.1	468	34.7		369	42.7	425	30.2	134	31.4	
												
**Education (age of termination)**					< 0.001							<0.001
No formal education	82	6.1	190	14.3		96	11.2	117	8.4	59	14.2	
< 15 years	627	46.7	582	43.9		445	51.8	593	42.5	171	41.2	
16–19 years	343	25.5	318	24.0		190	22.1	358	25.7	113	27.2	
>19 non-university	181	13.5	147	11.1		72	8.4	211	15.1	45	10.8	
University	110	8.2	89	6.7		56	6.5	116	8.3	27	6.5	
												
**Work status**					< 0.001							0.004
Workers, students or housewives	1178	87.3	1076	79.8		747	86.4	1155	82.1	352	82.4	
Unemployed or first-time job-seekers	104	7.7	161	11.9		57	6.6	161	11.5	47	11.0	
Retirees and pensioners	68	5.0	111	8.2		61	7.1	90	6.4	28	6.6	
												
**Occupation: fishing**					< 0.001							< 0.001
Yes	1	0.1	152	17.2		5	0.9	26	2.8	122	37.3	
No	937	98.5	718	81.4		566	97.9	892	96.1	197	60.2	
												
**Tobacco**					< 0.001							<0.001
Never smoker	851	63.0	667	49.4		548	63.6	793	56.4	177	42.0	
Ex-smoker	127	9.4	174	12.9		81	9.4	158	11.2	62	14.7	
Current smoker	366	27.1	502	37.2		232	26.9	454	32.3	182	43.2	
												
**Hours of sleep daily**					<0.001							0.002
< 7 hours	174	12.9	239	17.7		132	15.3	190	13.5	91	21.3	
7 – 9 hours	931	69.0	929	68.8		585	67.6	1001	71.09	274	64.2	
> 9 hours	245	18.1	182	13.5		148	17.1	217	15.4	62	14.5	
												
**Reported morbidity**												
Arterial hypertension	119	8.8	96	7.1	0.103	80	9.3	107	7.6	28	6.6	0.189
Hypercholesterolemia	135	10.0	125	9.3	0.518	94	10.9	126	9.0	40	9.4	0.322
Diabetes mellitus	31	2.3	51	3.8	0.025	32	3.7	34	2.4	16	3.8	0.147
Asthma or bronchitis	35	2.6	62	4.6	0.005	25	2.9	51	3.6	21	4.9	0.183
Heart diseases	35	2.6	37	2.8	0.809	22	2.5	40	2.9	10	2.3	0.819
Stomach ulcer	31	2.3	24	1.8	0.342	16	1.9	30	2.1	9	2.1	0.891
Allergy	99	7.4	126	9.4	0.059	56	6.5	122	8.7	47	11.0	0.017
Cancer	4	0.3	5	0.4	0.738	0	0.0	6	0.4	3	0.7	0.036
Anxiety, distress, nerves	102	7.6	94	7.0	0.557	63	7.3	107	7.6	26	6.1	0.568
Depression	61	4.5	78	5.8	0.137	52	6.0	68	4.9	19	4.5	0.364
Insomnia	62	4.6	58	4.3	0.712	46	5.3	58	4.1	16	3.8	0.305
												
**Residential exposure**												<0.001
Interior						689	79.7	648	46.0	13	3.0	
Coast						176	20.4	760	54.0	414	97.0	

In terms of individual exposure, men outnumbered women in the seriously affected group, and the most exposed segment comprised persons aged 30 to 44 years. Almost 60% of seriously exposed persons reported being current or ex-smokers. When it came to hours of sleep, it was the seriously affected who least frequently reported sleeping 7 to 9 hours per day. Insofar as reported morbidity was concerned, there was a higher prevalence of allergies among the seriously and slightly affected versus the unexposed group. Finally, while there were similar percentages of slightly affected persons in seaside and inland areas, unaffected persons came mainly from the interior (79.7%) and all seriously affected persons came from the coast (97.0%) (Table 1).

Prior to analyzing the scores of the respective questionnaires, the internal consistency indices (Cronbach's alpha) were calculated for each dimension of each questionnaire, and proved higher than 0.7 in all cases.

### Residential exposure

In general, the mean scores for the 8 dimensions of the SF-36 questionnaire were fairly similar in terms of residential exposure (Table 2). Nevertheless, small, statistically significant differences were in evidence, e.g., residents along the coast registered a better score than those in the interior for bodily pain (85.53 vs. 83.56; p = 0.029) but, in contrast, registered a worse score for general health (67.48 vs. 69.20; p = 0.025) and mental health (75.93 vs. 79.19; p < 0.001), with this being the only dimension in which the difference could be considered relevant.

**Table 2:** SF-36 means according to residential exposure

**Residential exposure**	**PF**		**RF**		**BP**		**GH**		**VT**		**SF**		**RE**		**MH**	
	Mean	(SD)	Mean	(SD)	Mean	(SD)	Mean	(SD)	Mean	(SD)	Mean	(SD)	Mean	(SD)	Mean	(SD)
								
Interior (n = 1350)	93.22	(14.16)	90.45	(27.13)	83.56	(23.80)	69.20	(18.89)	69.18	(19.27)	93.80	(15.59)	96.01	(16.74)	79.19	(17.27)
Coast (n = 1350)	93.45	(14.62)	90.25	(27.38)	85.53	(22.94)	67.48	(20.62)	68.77	(19.19)	93.45	(16.09)	94.85	(19.21)	75.93	(18.22)
p	*0.678*		*0.853*		*0.029*		*0.025*		*0.585*		*0.563*		*0.097*		*<0.001*	

PF, Physical functioning; RF, Role-physical; BP, Bodily pain; GH, General health; VT, Vitality; SF, Social functioning; RE, Role-emotional; MH, Mental health

Comparison of suboptimal and optimal SF-36 scores indicated that coastal subjects had less likelihood of scoring low in physical functioning (OR:0.69; 95%CI:0.54–0.89) and bodily pain (OR:0.74; 95%CI:0.62–0.91), but had a higher risk of registering suboptimal scores in mental health (OR:1.28; 95%CI:1.02–1.61) (Table 3). None of the GHQ-28 dimensions or HADS subscales showed statistically significant differences between the coast and the interior. Lastly, coastal residents registered a higher frequency of suboptimal values on the GADS depression subscale (OR:1.72; 95%CI:1.18–2.49) (Table 3).

**Table 3:** Health-related quality of life and mental health indicators according to residential exposure to the Prestige oil-spill

	**Interior (n = 1350)**		**Coast (n = 1350)**		**OR^1^**	**95% CI^1^**	**p**
		
	**N**	**%**	**N**	**%**			
					
**SF-36**							
**Physical functioning**							
Subjects with suboptimal scores	322	24.08	270	20.13	0.69	0.54 – 0.89	0.005
**Role-physical**							
Subjects with suboptimal scores	176	13.16	179	13.35	0.96	0.73 – 1.28	0.811
**Bodily pain**							
Subjects with suboptimal scores	537	40.16	496	36.99	0.74	0.62 – 0.91	0.003
**General health**							
Subjects with suboptimal scores	367	27.45	436	32.51	1.15	0.93 – 1.43	0.193
**Vitality**							
Subjects with suboptimal scores	173	12.94	167	12.45	0.85	0.63 – 1.13	0.271
**Social functioning**							
Subjects with suboptimal scores	263	19.67	275	20.51	1.08	0.86 – 1.38	0.501
**Role-emotional**							
Subjects with suboptimal scores	91	6.81	111	8.28	1.21	0.85 – 1.75	0.278
**Mental health**							
Subjects with suboptimal scores	266	19.90	340	25.35	1.28	1.02 – 1.61	0.036
**GHQ-28**							
**Somatic symptoms**							
Cases	28	2.07	37	2.75	1.48	0.82 – 2.68	0.196
**Anxiety and insomnia**							
Cases	31	2.30	43	3.20	1.07	0.60 – 1.91	0.762
**Severe depression**							
Cases	4	0.30	4	0.30	0.76	0.13 – 4.38	0.776
**Social dysfunction**							
Cases	12	0.89	21	1.56	1.91	0.82 – 4.42	0.133
**HADS**							
**Anxiety**							
Cases (prob+conf)*	133	9.85	148	10.96	0.97	0.71 – 1.32	0.836
Cases (conf)**	53	3.93	63	4.67	1.15	0.72 – 1.84	0.549
**Depression**							
Cases (prob+conf)*	36	2.67	46	3.41	1.17	0.67 – 2.06	0.581
Cases (conf)**	11	0.81	14	1.04	0.92	0.33 – 2.52	0.870
**GADS**							
**Anxiety**							
Cases	163	12.07	177	13.11	1.01	0.76 – 1.35	0.949
**Depression**							
Cases	85	6.30	126	9.33	1.72	1.18 – 2.49	0.004

1 OR: OR – 95% CI = odds ratio (coast versus interior) adjusted for individual exposure, age, sex, work status, education, smoking, hours of sleep daily and number of chronic diseases. -95% confidence interval.

* Probable (prob) and confirmed (conf) cases included.

** Only confirmed (conf) cases included.

### Individual exposure

We detected no substantial differences in SF-36 dimensions except for "physical functioning". Scores for this latter dimension rose with level of exposure (91.51 unaffected, 93.86 slightly affected and 95.28 seriously affected, p < 0.001) (Table 4).

**Table 4:** SF-36 means according to individual exposure

**Individual exposure**	**PF**		**RF**		**BP**		**GH**		**VT**		**SF**		**RE**		**MH**	
	Mean	(SD)	Mean	(SD)	Mean	(SD)	Mean	(SD)	Mean	(SD)	Mean	(SD)	Mean	(SD)	Mean	(SD)
								
Unaffected (n = 865)	91.51	(16.95)	88.79	(29.64)	83.97	(24.58)	67.61	(20.28)	68.55	(19.99)	92.87	(16.74)	95.13	(18.76)	78.14	(17.90)
Slightly affected (n = 1408)	93.86	(13.30)	91.05	(26.12)	84.61	(22.71)	68.85	(19.84)	69.08	(19.07)	93.92	(15.50)	95.66	(17.34)	77.44	(18.10)
Seriously affected (n = 427)	95.28	(11.59)	91.19	(25.77)	85.51	(23.19)	68.13	(18.61)	69.48	(18.15)	94.15	(15.04)	95.27	(18.75)	76.74	(16.69)
P	*<0.001*		*0.074*		*0.269*		*0.434*		*0.390*		*0.117*		*0.763*		*0.174*	

PF, Physical functioning; RF, Role-physical; BP, Bodily pain; GH, General health; VT, Vitality; SF, Social functioning; RE, Role-emotional; MH, Mental health

This association between SF-36 suboptimal scores for "physical functioning" and individual exposure no longer proved significant in the multivariate analysis, when seriously affected were compared to unexposed subjects (OR:0.93; 95%CI:0.63–1.38) (Table 5). Also, HADS depression scores improved very slightly as exposure increased (1.86 unaffected, 1.61 slightly affected, and 1.48 seriously affected, p = 0.002), though the adjusted OR failed to reach statistical significance. A striking result was the lower proportion of depression registered by seriously affected persons in the GADS questionnaire (OR: 0.47; 95%CI:0.26–0.85).

**Table 5:** Health-related quality of life and mental health indicators according to individual exposure to the *Prestige *oil-spill

	**Unaffected (n = 865)**		**Slightly affected (n = 1408)**		**OR^1^**	**95% CI^1^**	**p**	**Seriously affected (n = 427)**		**OR^2^**	**95% CI^2^**	**p**
			
	**N**	**%**	**N**	**%**				**N**	**%**			
**SF-36**												
**Physical functioning**												
S.W.S.S.^3^	220	25.70	299	21.37	0.99	0.77 – 1.26	0.909	73	17.26	0.93	0.63 – 1.38	0.073
**Role-physical**												
S.W.S.S.^3^	122	14.25	180	12.87	0.92	0.69 – 1.22	0.562	53	12.53	0.93	0.61 – 1.44	0.774
**Bodily pain**												
S.W.S.S.^3^	320	37.38	559	39.96	1.29	1.05 – 1.57	0.013	154	36.41	1.29	0.95 – 1.74	0.098
**General health**												
S.W.S.S.^3^	260	30.37	414	25.59	1.00	0.80 – 1.25	0.986	129	30.50	0.96	0.69 – 1.32	0.789
**Vitality**												
S.W.S.S.^3^	121	14.14	173	12.37	0.88	0.65 – 1.18	0.388	46	10.87	0.92	0.59 – 1.45	0.725
**Social functioning**												
S.W.S.S.^3^	189	22.08	273	19.51	0.79	0.62 – 1.00	0.055	76	17.97	0.74	0.51 – 1.06	0.104
**Role-emotional**												
S.W.S.S.^3^	68	7.94	104	7.43	0.86	0.59 – 1.23	0.408	30	7.09	0.80	0.46 – 1.38	0.417
**Mental health**												
S.W.S.S.^3^	180	21.30	330	23.59	1.06	0.83 – 1.35	0.631	96	22.70	1.00	0.70 – 1.43	0.993
**GHQ-28**												
**Somatic Symptoms**												
Cases	27	3.12	29	2.07	0.60	0.34 – 1.09	0.093	9	2.12	0.61	0.25 – 1.47	0.268
**Anxiety and insomnia**												
Cases	20	2.31	40	2.85	0.10	0.60 – 2.01	0.762	14	3.29	1.36	0.59 – 3.16	0.472
**Severe depression**												
Cases	2	0.23	5	0.36	1.35	0.21 – 8.55	0.752	1	0.24	1.05	0.06 – 18.96	0.973
**Social dysfunction**												
Cases	11	1.27	18	1.28	0.87	0.37 – 2.07	0.755	4	0.94	0.59	0.16 – 2.21	0.432
**HADS**												
**Anxiety**												
Cases (prob+conf)*	87	10.06	150	10.65	1.04	0.76 – 1.44	0.786	44	10.3	1.14	0.71 – 1.86	0.576
Cases (conf)**	39	4.51	59	4.19	0.85	0.53 1.36	0.496	18	4.22	0.81	0.39 – 1.67	0.572
**Depression**												
Cases (prob+conf)*	33	3.82	40	2.84	0.67	0.38 – 1.16	0.150	9	2.11	0.57	0.21 – 1.33	0.176
Cases (conf)*	10	1.16	11	0.78	0.86	0.32 – 2.32	0.771	4	0.94	1.60	0.38 – 6.78	0.527
**GADS**												
**Anxiety**												
Cases	112	12.95	179	12.71	0.92	0.69 – 1.24	0.602	49	11.48	0.88	0.56 – 1.38	0.582
**Depression**												
Cases	75	8.67	110	7.81	0.71	0.48 – 1.03	0.071	26	6.09	0.47	0.26 – 0.85	0.012

1 OR: OR – 95% CI = odds ratio (slightly affected versus unaffected) adjusted for residential exposure, age, sex, work status, education, smoking, hours of sleep daily and number of chronic diseases -95% confidence interval.

2 OR: OR – 95% CI = odds ratio (seriously affected versus unaffected) adjusted for residential exposure, age, sex, work status, education, smoking, hours of sleep daily and number of chronic diseases. -95% confidence interval.

3 Subjects with suboptimal scores

* Probable (prob) and confirmed (conf) cases included.

** Only confirmed (conf) cases included

## Discussion

This paper presents the results of a large epidemiologic study designed to assess the possible effects of the *Prestige *oil-spill on the HRQoL and mental health of residents of affected towns and villages. Although no SF-36 scores are available for the preceding period in these areas, the SF-36 scores hardly differ from the normative population values in Spain [31]. Moreover, there are few differences in HRQoL in terms of exposure, whether residential or personal, to the *Prestige *oil-spill. The only results that would suggest a possible negative impact are the worse scores for the mental health dimension of the SF-36 questionnaire obtained by residents in the most exposed area, and their greater risk of being defined as a case in the GADS depression scale. On the other hand, the better scores in the physical dimensions of HRQoL associated with individual exposure, might be explained by the exposure criteria, since the professional and leisure activities that determine a person's classification as "exposed" require a certain degree of physical health.

For comparison purposes, we would have preferred to select Galician coastal towns that were not affected by the Prestige spill, but the unaffected coastal areas of Galicia displayed substantial demographic and economic differences. Tourism and industry are the main economic activities along the unaffected stretch of the Galician coast (*Rías Bajas*), yet these activities play a minor role in the overall economy of the affected area. Consequently, the reference group was made of neighboring rural towns in the interior, which had demographic and economic indicators that were more similar to those of the affected area.

For proper interpretation of our results, account should be taken of the time elapsed between the oil-spill and data-collection, since the interviews were held almost one and a half years after the first oil washed ashore. Hence, some of those affected may have benefited from individual compensation or from official government policy to foster the economic recovery of the affected areas (*Plan Galicia*). The influence of such aid on subjects' perception of health, physical and psychological, could not be investigated by this study, since we did not obtain information on the aid payments received by participants. Similarly, information on personal stressful events which might have had a negative influence on interviewees' perception of health was also unavailable. Finally, though selective non-response is within the bounds of possibility, only 11% of subjects included in the original sample refused to participate in the study. Two thirds of nonparticipants in the original sample were not included due to a lack of accuracy in the municipal rolls or repeated intractability, possibly indicating that these persons were not living in the area at the time when the study was conducted. We attempted to counteract these losses by selecting two other randomized samples so as to replace the original candidate with a randomly selected substitute.

The use of the various instruments allowed for measurement of different dimensions of health. Whilst the GADS and HADS questionnaires solely furnish information on mental health, the SF-36 and GHQ-28 enable dimensions other than the mental, such as physical and social, to be explored.

In mental health, differences between the coast and the interior were detected by the SF-36 and GADS, though comparable dimensions in the remaining questionnaires showed no association. In HRQoL, greater problems are posed by evaluation of psychological versus physical dimensions, because the former are more subjective and less easily observable. Indeed, when HRQoL questionnaires are answered by patients and proxy respondents, concordance between the respective results is good in the case of the physical dimensions but decreases in the case of psychological and social dimensions [32-34]. Furthermore, while the questionnaires used address psychological dimensions of HRQoL, they use different approaches, i.e., whereas the SF-36 inquires into general aspects of mental health and the GADS includes questions linked to somatic symptoms, insomnia, self-confidence, and vitality in its subscales, the HADS and GHQ-28 questionnaire inquire into more specific symptoms of severe anxiety and depression. This could explain the greater concordance between the results of the SF-36 and GADS, and the differences vis-à-vis the others.

The exposure criteria considered are interrelated: indeed, while almost all seriously affected persons came from the coast, unaffected persons mainly (80%) came from the interior. When individual exposure was considered, no impact on mental health was observed with the different questionnaires. However, for residential exposure, a negative association between exposure and depression was found with the SF-36 and GADS questionnaires. This discrepancy in the results on considering ecologic (area of residence) and individual exposure could reflect differential nuances in the two classifications. In order to distinguish between individual and ecologic effects, a further analysis was carried out in which exposure was divided into three categories, namely: a) residential; b) individual; and, c) both types of exposures. The results are provided as supplementary information [see Additional file 2]. The most interesting result in this analysis is the higher prevalence of anxiety and depression found with the GADS questionnaire among persons who were not individually exposed but lived in the affected area. However, the number of subjects only residentially exposed is small and it is difficult to extract conclusions from this analysis. It should be borne in mind that the two types of exposure, though interrelated, are different. Individual exposure is determined by work or leisure activities that entail direct contact with or indirect affection by the oil-spill. Subjects involved in clean-up tasks and those affected in their occupational activity are the economically active population, thereby entailing the possibility of a healthy-worker bias (because the exposed group is physically healthier than the comparison group). Moreover, the consequences of the catastrophe in this group are essentially economic. Residential exposure, on the other hand, reflects the influence of the oil-spill on the setting in which subjects undertake their everyday activities, and its emotional impact thus goes far beyond its purely financial or commercial scope.

Some of the studies conducted in the wake of other huge oil-spills off coastal areas show a negative impact on the different dimensions of HRQoL among the affected population. After the *Exxon Valdez *accident (Alaska, 1989), exposed subjects presented with a higher frequency of anxiety, post-traumatic stress, and depression (data-collection carried out one year after the spill) [35]. Residents in areas affected by the sinking of the *Braer *(Scotland, 1993) registered worse subjective health and more psychological disorders than did residents in unexposed areas (data-collection carried out six month after the spill) [36]. Following the foundering of the *Sea Empress *(Wales, 1996), the inhabitants of coastal towns registered a greater frequency of anxiety and depression, and worse levels of mental health than did inland residents (data-collection carried out four weeks after the spill) [37]. Finally, after the *Tasman Spirit *oil-spill (Pakistan, 2003), residents in areas close to the accident registered a possible association between acute health problems and exposure, indicating adverse effects on their health (data-collection carried out three weeks after the spill) [38]. In our case, it seems that sixteen months after the spill the impact on mental health was minimal or non-existent.

Although the similarity between the above-mentioned accidents and the *Prestige *is evident, it is interesting to consider some differential aspects. First, experience of oil-spills is unfortunately nothing new in the affected area. Second, whereas oil-spills usually take place at a specific moment in time and over a relatively short period, the *Prestige *continued losing oil for more than three months after it sank [39]. Lastly, for months after the accident, the towns affected continued to receive hundreds of volunteers who took part in the clean-up operation, thereby possibly adding a positive aspect to the disaster.

The differences between the results reported by this study and those of similar accidents are thus evident. Moreover, comparisons between these types of disasters are difficult, not only because of the different periods and forms of exposure, but also because of the psychological and social differences that characterize the victims [35]. Despite the fact that there is little empirical evidence as to the role played by social aid in the process of post-disaster recovery, the importance of such aid must be borne in mind when it comes to understanding the results of this type of study [40].

With regard to the economic impact on the affected population, there are substantial differences between the *Prestige *and previous accidents of this type in Galiza. While 10 to 15 years had had to pass before compensation for previous oil-spills was forthcoming [41], fishermen, shellfishers and shipowners affected by the ban on fishing after the *Prestige *spill waited a little over one month before they started receiving compensatory payments, arguably linked to the greater social, political and media repercussion generated by the *Prestige *accident. By December 31, 2002, close to 24 million euros had already been paid out in the form of aid [42]. One year later, over 114 million euros had been received by the Galician fishing sector [43]. In addition, all the towns along the *Costa da Morte *(literally, "Coast of Death"), heavily affected by the spill, were included in the Galician Ports & Harbors Plan (*Plan Galicia de Puertos *– 42.3 million euros) [44]. Compensation and temporary jobs deriving from the clean-up and from implementation of the Galician Ports & Harbors Plan have probably mitigated the financial component of the disaster, rendering it possible for fishermen and other professionals affected to have a stable income whilst their professional activity was at a halt. Hence, whereas some populations affected by other oil-spills, such as that which happened in Alaska, waited years before receiving the relevant indemnities [14], the towns studied here were quick to receive, not merely the corresponding financial aid, but also important social support in the form of the thousands of volunteers who rallied to participate in the clean-up.

## Conclusion

In conclusion, almost one and half years after the ecologic catastrophe that struck the Galician coast, worse HRQoL and mental health levels were not in evidence among subjects who were personally affected by the oil-spill or among those who, regardless of their individual exposure, resided in towns and villages whose shorelines had suffered severe oil pollution. In the medium term, however, results for some of the scales used might indicate a slight impact of the oil-spill on the mental health of residents in the affected areas.

## Competing interests

The author(s) declare that they have no competing interests.

## Authors' contributions

JMC conceived the idea, carried out the statistical analysis and wrote the manuscript. BPG, MJG, VL, NA, MJF, PGC and GLA made contribution to statistical analyses and interpretation of results, and revised the manuscript for important intellectual content. FRA and MP designed the study, contributed to manuscript writing, and revised it for important intellectual content. All authors contributed to the final version of the manuscript.

**Figure 1:**
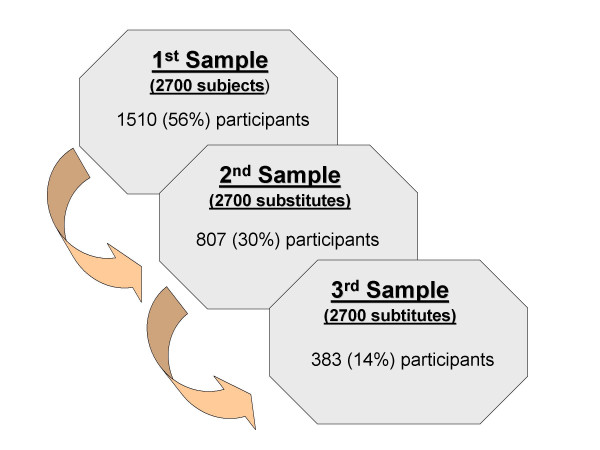
Sampling design. Two substitutes, with similar characteristics, were assigned to each subject in the first sample.

## Pre-publication history

The pre-publication history for this paper can be accessed here:



## Supplementary Material

Additional file 1 Sampling design. This figure presents the sampling design. Click here for file
