# Supplementary material for: Health-related quality of life and mental health in the medium-term aftermath of the Prestige oil spill in Galiza (Spain): a cross-sectional study
Source: BMC Public Health. 2007 Sep 17;7:245. doi: 10.1186/1471-2458-7-245 (PMC2194772; doi:10.1186/1471-2458-7-245)
Supplement: Additional file 1 — Sampling design. This figure presents the sampling design. [file 1471-2458-7-245-S1.doc]

Analysis comparing people with different types of oil-spill exposure: 1) only residentially exposed, 2) only individually exposed, 3) people with both types of exposure. Unaffected subjects are taken as reference.

|  |  | **RESIDENTIAL (n=176)** | | | | |  | **INDIVIDUAL (n=661)** | | | | |  | **RESIDENTIAL AND INDIVIDUAL**  **(n=1174)** | | | | |
| --- | --- | --- | --- | --- | --- | --- | --- | --- | --- | --- | --- | --- | --- | --- | --- | --- | --- | --- |
|  |  | **OR^1^** | **95% IC**^1^ | | | **p** |  | **OR^2^** | **95% IC**^2^ | | | **p** |  | **OR^1^** | **95% IC**^3^ | | | **P** |
|  |  |  |  |  |  |  |  |  |  |  |  |  |  |  |  |  |  |  |
| SF-36 |  |  |  |  |  |  |  |  |  |  |  |  |  |  |  |  |  |  |
| Physical functioning |  |  |  |  |  |  |  |  |  |  |  |  |  |  |  |  |  |  |
| S. suboptimal scores.^4^ |  | 0.84 | 0.54 | - | 1.32 | 0.454 |  | 1.07 | 0.80 | - | 1.43 | 0.658 |  | 0.68 | 0.52 | - | 0.89 | 0.005 |
| Role physical |  |  |  |  |  |  |  |  |  |  |  |  |  |  |  |  |  |  |
| S. suboptimal scores. ^4^ |  | 1.52 | 0.95 | - | 2.43 | 0.083 |  | 1.15 | 0.82 | - | 1.62 | 0.421 |  | 0.93 | 0.69 | - | 1.27 | 0.665 |
| **Bodily pain** |  |  |  |  |  |  |  |  |  |  |  |  |  |  |  |  |  |  |
| S. suboptimal scores. ^4^ |  | 0.66 | 0.45 | - | 0.96 | 0.028 |  | 1.22 | 0.97 | - | 1.54 | 0.089 |  | 0.95 | 0.77 | - | 1.17 | 0.639 |
| **General health** |  |  |  |  |  |  |  |  |  |  |  |  |  |  |  |  |  |  |
| S. suboptimal scores. ^4^ |  | 1.29 | 0.87 | - | 1.92 | 0.202 |  | 1.05 | 0.81 | - | 1.37 | 0.703 |  | 1.15 | 0.91 | - | 1.45 | 0.239 |
| **Vitality** |  |  |  |  |  |  |  |  |  |  |  |  |  |  |  |  |  |  |
| S. suboptimal scores. ^4^ |  | 1.10 | 0.67 | - | 1.82 | 0.704 |  | 1.00 | 0.70 | - | 1.41 | 0.981 |  | 0.77 | 0.56 | - | 1.06 | 0.115 |
| **Social functioning** |  |  |  |  |  |  |  |  |  |  |  |  |  |  |  |  |  |  |
| S. suboptimal scores. ^4^ |  | 0.83 | 0.54 | - | 1.29 | 0.410 |  | 0.70 | 0.52 | - | 0.94 | 0.016 |  | 0.82 | 0.64 | - | 1.06 | 0.124 |
| **Role emotional** |  |  |  |  |  |  |  |  |  |  |  |  |  |  |  |  |  |  |
| S. suboptimal scores. ^4^ |  | 1.63 | 0.90 | - | 2.95 | 0.107 |  | 1.01 | 0.64 | - | 1.59 | 0.971 |  | 1.06 | 0.71 | - | 1.59 | 0.779 |
| **Mental health** |  |  |  |  |  |  |  |  |  |  |  |  |  |  |  |  |  |  |
| S. suboptimal scores. ^4^ |  | 1.36 | 0.89 | - | 2.08 | 0.153 |  | 1.09 | 0.81 | - | 1.46 | 0.559 |  | 1.34 | 1.04 | - | 1.74 | 0.025 |
|  |  |  |  |  |  |  |  |  |  |  |  |  |  |  |  |  |  |  |
| **GHQ-28** |  |  |  |  |  |  |  |  |  |  |  |  |  |  |  |  |  |  |
| Somatic symptoms |  |  |  |  |  |  |  |  |  |  |  |  |  |  |  |  |  |  |
| Cases |  | 1.84 | 0.79 | - | 4.30 | 0.157 |  | 0.73 | 0.33 | - | 1.60 | 0.433 |  | 0.92 | 0.48 | - | 1.75 | 0.798 |
| Anxiety and insomnia |  |  |  |  |  |  |  |  |  |  |  |  |  |  |  |  |  |  |
| Cases |  | 2.72 | 1.04 | - | 7.08 | 0.041 |  | 1.86 | 0.86 | - | 4.03 | 0.113 |  | 1.53 | 0.75 | - | 3.16 | 0.245 |
| **Severe depression** |  |  |  |  |  |  |  |  |  |  |  |  |  |  |  |  |  |  |
| Cases |  | - | - | - | - | - |  | 0.92 | 0.12 | - | 7.28 | 0.935 |  | 0.87 | 0.13 | - | 5.74 | 0.882 |
| **Social dysfunction** |  |  |  |  |  |  |  |  |  |  |  |  |  |  |  |  |  |  |
| Cases |  | 2.49 | 0.65 | - | 9.49 | 0.183 |  | 1.06 | 0.33 | - | 3.40 | 0.927 |  | 1.58 | 0.59 | - | 4.23 | 0.359 |
|  |  |  |  |  |  |  |  |  |  |  |  |  |  |  |  |  |  |  |
| **H.A.D.S.** |  |  |  |  |  |  |  |  |  |  |  |  |  |  |  |  |  |  |
| Anxiety |  |  |  |  |  |  |  |  |  |  |  |  |  |  |  |  |  |  |
| Cases (prob+conf)* |  | 1.66 | 0.97 | - | 2.86 | 0.065 |  | 1.36 | 0.92 | - | 2.02 | 0.121 |  | 1.12 | 0.78 | - | 1.60 | 0.553 |
| Cases (conf)** |  | 1.81 | 0.85 | - | 3.87 | 0.127 |  | 1.09 | 0.61 | - | 1.94 | 0.781 |  | 1.02 | 0.60 | - | 1.74 | 0.940 |
| **Depression** |  |  |  |  |  |  |  |  |  |  |  |  |  |  |  |  |  |  |
| Cases (prob+conf) |  | 1.27 | 0.54 | - | 2.98 | 0.578 |  | 0.71 | 0.34 | - | 1.45 | 0.344 |  | 0.74 | 0.40 | - | 1.37 | 0.337 |
| Cases (conf) |  | 1.76 | 0.41 | - | 7.60 | 0.452 |  | 1.38 | 0.39 | - | 4.84 | 0.618 |  | 1.06 | 0.33 | - | 3.43 | 0.925 |
|  |  |  |  |  |  |  |  |  |  |  |  |  |  |  |  |  |  |  |
| **G.A.D.S.** |  |  |  |  |  |  |  |  |  |  |  |  |  |  |  |  |  |  |
| **Anxiety** |  |  |  |  |  |  |  |  |  |  |  |  |  |  |  |  |  |  |
| Cases |  | 1.69 | 1.03 | - | 2.75 | 0.036 |  | 1.20 | 0.84 | - | 1.71 | 0.326 |  | 0.98 | 0.70 | - | 1.35 | 0.882 |
| **Depression** |  |  |  |  |  |  |  |  |  |  |  |  |  |  |  |  |  |  |
| Cases |  | 2.44 | 1.35 | - | 4.39 | 0.003 |  | 0.89 | 0.55 | - | 1.46 | 0.655 |  | 1.14 | 0.75 | - | 1.75 | 0.543 |

^1^ OR: OR – 95% CI = odds ratio (only residentially exposed versus unaffected) adjusted for age, sex, work status, education, smoking, hours of sleep daily and number of chronic diseases – 95% confidence interval.

^2^ OR: OR – 95% CI = odds ratio (only individually exposed versus unaffected) adjusted for age, sex, work status, education, smoking, hours of sleep daily and number of chronic diseases – 95% confidence interval.

^3^ OR: OR – 95% CI = odds ratio (both types of exposure versus unaffected) adjusted for age, sex, work status, education, smoking, hours of sleep daily and number of chronic diseases – 95% confidence interval.

^4^ Subjects with suboptimal scores.

^*^ Probable (prob) and confirmed (conf) cases included.

^**^ Only confirmed (conf) cases included
